# Knowledge, attitude and practice among pharmacy students and faculty members towards artificial intelligence in pharmacy practice: A multinational cross-sectional study

**DOI:** 10.1371/journal.pone.0296884

**Published:** 2024-03-01

**Authors:** Hisham E. Hasan, Deema Jaber, Samaa Al Tabbah, Nabih Lawand, Hana A. Habib, Noureldin M. Farahat

**Affiliations:** 1 Department of Clinical Pharmacy, Faculty of Pharmacy, Zarqa University, Zarqa, Jordan; 2 School of Pharmacy, Lebanese American University, Beirut, Lebanon; 3 Department of Psychology, Faculty of Medicine, Beirut Arab University, Beirut, Lebanon; 4 Department of Pharmaceutics, Faculty of Pharmacy, Benghazi University, Benghazi, Libya; 5 Department of Clinical Pharmacy, Faculty of Pharmacy, Alexandria University, Alexandria, Egypt; UCSI University, MALAYSIA

## Abstract

**Background:**

Modern patient care depends on the continuous improvement of community and clinical pharmacy services, and artificial intelligence (AI) has the potential to play a key role in this evolution. Although AI has been increasingly implemented in various fields of pharmacy, little is known about the knowledge, attitudes, and practices (KAP) of pharmacy students and faculty members towards this technology.

**Objectives:**

The primary objective of this study was to investigate the KAP of pharmacy students and faculty members regarding AI in six countries in the Middle East as well as to identify the predictive factors behind the understanding of the principles and practical applications of AI in healthcare processes.

**Material and methods:**

This study was a descriptive cross-sectional survey. A total of 875 pharmacy students and faculty members in the faculty of pharmacy in Jordan, Palestine, Lebanon, Egypt, Saudi Arabia, and Libya participated in the study. Data was collected through an online electronic questionnaire. The data collected included information about socio-demographics, understanding of AI basic principles, participants’ attitudes toward AI, the participants’ AI practices.

**Results:**

Most participants (92.6%) reported having heard of AI technology in their practice, but only a small proportion (39.5%) had a good understanding of its concepts. The overall level of knowledge about AI among the study participants was moderate, with the mean knowledge score being 42.3 ± 21.8 out of 100 and students having a significantly higher knowledge score than faculty members. The attitude towards AI among pharmacy students and faculty members was positive, but there were still concerns about the impact of AI on job security and patient safety. Pharmacy students and faculty members had limited experience using AI tools in their practice. The majority of respondents (96.2%) believed that AI could improve patient care and pharmacy services. However, only a minority (18.6%) reported having received education or training on AI technology. High income, a strong educational level and background, and previous experience with technologies were predictors of KAP toward using AI in pharmacy practice. Finally, there was a positive correlation between knowledge about AI and attitudes towards AI as well as a significant positive correlation between AI knowledge and overall KAP scores.

**Conclusion:**

The findings suggest that while there is a growing awareness of AI technology among pharmacy professionals in the Middle East and North Africa (MENA) region, there are still significant gaps in understanding and adopting AI in pharmacy Practice.

## Introduction

The simulation of human intellect by computers is known as artificial intelligence (AI) [1]. Collecting data, developing rules for interpreting the information, making approximate conclusions, and self-correction are all part of the process [2]. Over the past ten years, AI has become increasingly popular in most aspects of modern life as people have come to see the value of AI-powered tools in the development of next-generation healthcare technology [3, 4].

In pharmacy, it has already started to have an impact on disciplines like drug discovery [5], drug design [6], drug delivery [7], and pharmacy practice [8], particularly in developed countries. However, it has been projected that parallel advancements in information technology and AI will revolutionize global health in low- and middle-income nations [9].

Generally speaking, pharmacists have concentrated their efforts on highlighting the importance of pharmacist-patient interaction [10, 11]. AI-driven solutions are altering the way pharmacists provide services and enhance results, from prescription management to drug development. These outcomes may be reached by establishing solid pharmacy practice standards and good technological knowledge in order to deliver the best pharmaceutical care services for patients [12, 13]. In order to successfully interact with data scientists to construct models that will enhance patient care, pharmacists and academics will need to understand the language and procedures utilized in AI [14–16].

Pharmacy students and faculty members are becoming increasingly knowledgeable about the potential applications of AI in pharmacy practice. Through their educational and training programs, they are introduced to the idea of AI [17]. They could learn about AI-powered innovations like robotic automation in dispensing procedures, predictive analytics for medication interactions, computer-aided drug creation, and intelligent decision support systems. However, depending on the curriculum and exposure offered by their individual universities, the breadth and depth of their knowledge may differ [18].

Regarding the attitude towards AI in pharmacy practice, it varies among pharmacy students and faculty members [19]. Some individuals may support the use of AI technologies and perceive their potential to advance patient care, enhance medication safety, and streamline pharmacy practice procedures. They could think of AI as a useful tool for streamlining drug therapy and minimizing pharmaceutical mistakes [20]. On the other side, some people could be wary or cautious about AI. This may be because they are worried about their job security, concerned about how difficult it will be to install AI systems, or unfamiliar with the technology. Personal experiences, exposure to real-world AI applications, and the degree of faith in AI algorithms and models can all have an impact on attitudes toward AI [21, 22].

The use of AI in pharmacy practice is still in its early stages, and various institutions may actually apply AI technology differently [23]. While some pharmacy schools or healthcare organizations may have limited exposure or access to AI-driven practices, others may actively incorporate AI into their practice settings [24, 25]. Knowing more about AI and having a favorable attitude towards it increases the likelihood that pharmacy students and faculty members will research and use it in their practice. However, the adoption of AI in pharmacy practice is dependent on various factors, including resource availability, training opportunities, regulatory considerations, technological infrastructure, and institutional support [26, 27].

AI integration in pharmacy practice has the potential to revolutionize the industry by boosting operational effectiveness, patient care, and drug management [28]. Understanding the knowledge, attitudes, and practices towards AI in pharmacy practice is important to effectively take full advantage of its benefits and resolve any concerns or challenges associated with its implementation [29]. To the best of our knowledge, our review revealed a scarcity of regionally or locally focused studies in this context. In light of the limited existing literature on this topic, we aim to establish a foundational dataset on pharmacists’ receptivity and perspectives regarding AI technology adoption, along with a comprehensive understanding of its integration in the field of pharmacy practice.

The primary objective of this study was to investigate the knowledge, attitudes, and practices (KAP) of pharmacy students and faculty members regarding AI in six countries in the Middle East (Jordan, Palestine, Lebanon, Egypt, Saudi Arabia, and Libya). The secondary objective was to identify the predictive factors behind the understanding of the principles and practical applications of AI in healthcare processes. By evaluating the present status of AI in pharmacy practice, we can gain valuable knowledge about how pharmacists perceive and welcome this emerging technology, ultimately shaping its integration into daily workflows.

## Material and methods

### Study design and participants

This was a descriptive cross-sectional study carried out from June 2022 to January 2023 to assess the knowledge, attitude, and practice among pharmacy students and faculty members towards artificial intelligence in pharmacy practice in Jordan, Palestine, Lebanon, Egypt, Saudi Arabia, and Libya. The study included a total of 875 participants from six countries, divided into 702 students at the Faculty of Pharmacy and 173 faculty members at the Faculty of Pharmacy.

### Sample size

The sample size was determined by an online sample size calculator (Raosoft^®^; Raosoft, Inc., United States). Considering the population in each requested country, the sample size was calculated by determining a margin of error of 5%, a confidence level of 95%, and a 50% response distribution.

### Inclusion-exclusion criteria and quality control

Participants included in the study were limited to individuals who were pharmacy students or faculty members from the six countries mentioned. They were required to provide informed consent and express their willingness to complete the survey. Individuals who did not meet these specified criteria were excluded from the study. To ensure data quality and reliability, several measures were implemented. The survey platform incorporated mandatory response fields to reduce missing data. The internal consistency and reliability of survey items were evaluated using Cronbach’s alpha coefficient, and items with low reliability were refined or removed. Additionally, duplicate or inconsistent responses were identified through logical checks embedded within the survey tool. Data cleaning procedures were performed to rectify any errors or inconsistencies in the dataset. To mitigate selection bias and enhance the representativeness of the sample, a systematic sampling approach was employed to recruit participants from various educational institutions and regions. In this approach, we employed a method of selecting universities at regular intervals from predefined lists of pharmacy faculties in the respective countries. The survey administration process was supervised by a research team, ensuring adherence to standardized protocols and ethical considerations.

### Data collection methods, instruments used, and measurements assessed

Data was collected through an online electronic questionnaire distributed via several methods, such as: e-mail, social media platforms (Facebook^®^, WhatsApp^®^, and LinkedIn^®^), or face-to-face. Furthermore, an announcement along with the questionnaire’s link was posted on targeted Facebook groups that belong to pharmacy students across universities. A reminder face-to-face visit was conducted to the target population at a number of universities every two months over a seven-month period to increase the response rate. Participation was completely voluntary, and data collection was entirely anonymous. After they were instructed about the nature and purpose of the survey, all respondents provided informed consent and were given the option to withdraw at any time.

The questionnaire was developed based on the objectives of the study and through a review of the literature [8, 14, 15, 30]. It underwent content validity testing by a pharmacy faculty member with expertise in pharmacy practice research and questionnaire development. Several modifications were made to the first draft of the questionnaire through an iterative process. The pre-final version of the questionnaire was uploaded and designed on Google Forms^®^, which is an electronic tool for developing online surveys [31]. The questionnaire was then piloted with a small group of pharmacy students and faculty members to test its clarity and comprehension, and minor modifications were made to produce the final version. The survey was originally designed in English, the official language of pharmacy education. However, recognizing the diverse academic levels of pharmacy students, we also provided a translated version in Arabic, ensuring that it was presented in a clear and unambiguous manner to accommodate responses from a broader range of participants.

The valid questionnaire consisted of four sections: (1) socio-demographic section; (2) knowledge section; (3) attitude section; and (4) practice section. All questions were closed-ended and answered by multiple choices or using a five-point Likert scale (strongly disagree, somewhat disagree, neutral, somewhat agree, strongly agree). The first section aimed to gather general demographic data including age, gender, country of residence, citizenship, monthly household net income (in each country’s currency), which was then classified based on World Bank data [32], student category (BPharm, PharmD, and MPharm), year of study, and self-reported tech-savviness (well informed about or proficient in the use of modern technology, especially computers). The second section explored the understanding of AI basic principles, advantages, disadvantages, and applications in general and during the COVID-19 pandemic. The third section aimed to assess participants’ attitudes toward AI, exploring whether the participant: (1) perceives AI as a partner or a competitor; (2) believes that healthcare providers will be replaced in the foreseeable future; (3) is frightened or excited by the developments; and (4) thinks that AI will improve pharmacy practice and would like it to be incorporated during their pharmacy study. The fourth section assessed the participants’ practice through the most frequently used references, sources of information, applications, and challenges related to using AI in pharmacy practice.

### Ethical considerations

The ethical integrity of this investigation adhered to the principles outlined in the World Medical Association’s Declaration of Helsinki. Ethical approval for the study was formally obtained from the institutional review board (IRB) committee of the Clinical Pharmacy Department and the Scientific Research Ethics Committee at Zarqa University (Approval No. 54/2021/2022). Participants provided written informed consent and participated on a voluntary basis. A comprehensive description of the study’s objectives was provided to participants before their engagement, focusing on the safeguarding of privacy. The study deliberately refrained from collecting any personally identifiable information, ensuring a robust level of participant anonymity. Additionally, participants were granted the option to discontinue their participation at any point during the survey. A stringent access control mechanism was implemented to guarantee the security and confidentiality of the study’s collected data.

### Statistical analysis

Statistical analyses of the collected data were performed using the Statistical Package for Social Sciences, version 27 (IBM SPSS^®^ Statistics for Windows; IBM Corp., United States). Descriptive and inferential statistics were used for the data analyses. Frequencies and percentages were used to summarize the responses generated. Data is presented as mean ± standard deviation (SD) or counts (%), as appropriate. KAP scores were calculated by summing responses for individual items, dividing by the number of items included in each section, and multiplying by 100. The *chi-*square test, independent *t-*test, ANOVA test, and Pearson’s *r* test were utilized to determine any significant differences among the study groups. Group comparisons between pharmacy students and faculty members were presented in tables. A *p-*value of less than 0.05 indicates statistical significance.

## Results

The study included a total of 875 participants from six countries: The majority were from Jordan (N = 296, 33.8%), followed by Egypt (N = 164, 18.7%), Lebanon (N = 163, 18.6%), and Libya (N = 161, 18.4%), while Palestine (N = 76, 8.7%), and Saudi Arabia (N = 15, 1.7%) made up the smallest proportion of the sample.

The demographic characteristics of students and faculty members participating in the study are presented in Table 1. The majority of participants were students at the Faculty of Pharmacy (N = 702, 80.2%), and the remaining (N = 173, 19.8%) were faculty members at the Faculty of Pharmacy. The majority of the respondents were 25.8 ± 9.4 years old, single (661, 75.5%), female (615, 70.3%), local citizens (780, 89.1%), governmental university affiliated (492, 56.2%), of upper-class net income (352, 40.2%), and mostly self-rated themselves as being neutrally tech-savvy (322, 36.8%).

**Table 1:** Demographic characteristics of students and faculty members.

Variable		Students N = 702	Faculty Members N = 173
**Age (Years)**		21.9 ± 2.9	41.5 ± 10.2
**Gender**	**Male**	191 (27.2%)	69 (39.9%)
	**Female**	511 (72.8%)	104 (60.1%)
**Citizenship**	**Local**	617 (87.9%)	163 (94.2%)
	**International**	85 (12.1%)	10 (5.8%)
**Material Status**	**Single**	624 (88.9%)	37 (21.4%)
	**Married**	54 (7.7%)	128 (74.0%)
	**Others**	24 (3.4%)	8 (4.6%)
**Monthly Household Income**	**Lower Class**	203 (28.9%)	20 (11.6%)
	**Middle Class**	257 (36.6%)	43 (24.9%)
	**Upper Class**	242 (34.5%)	110 (63.6%)
**University**	**Governmental**	390 (55.6%)	102 (59.0%)
	**Private**	312 (44.4%)	71 (41.0%)
**Work Place** ^***a***^	**Pharmacy**	148 (77.1%)	0 (0.0%)
	**Hospital**	23 (12.0%)	0 (0.0%)
	**University**	0 (0.0%)	173 (100.0%)
	**Others**	21 (10.9%)	0 (0.0%)
	**Missing**	59	0
**Tech-savvy**	**Strongly Disagree**	41 (5.8%)	9 (5.2%)
	**Disagree**	115 (16.4%)	25 (14.5%)
	**Neutral**	267 (38.0%)	55 (31.8%)
	**Agree**	166 (23.6%)	57 (32.9%)
	**Strongly Agree**	113 (16.1%)	27 (15.6%)

^*a*^This question allows for multiple responses.

The distribution of pharmacy students is presented in Table 2. Of the 702 students who responded to the survey, (374, 53.3%) were BPharm students, while (308, 43.9%) and (20, 2.8%) were PharmD students and MPharm students, respectively. The number of BPharm students who responded was higher than that of PharmD students. However, no significant differences were observed between the responses provided by both groups. The majority of students attended public school (452, 64.4%), are currently in their fourth year (256, 36.5%), have a very good level of achievement (334, 47.6%), and (451, 64.2%) are unemployed. All the reported differences in the distribution of students across the majors were statistically significant (*p* < 0.05).

**Table 2:** The distribution of pharmacy students.

Variable		Total Students N = 702	Current Major (Students)			*p-*value^*a*^
			Bachelor of Pharmacy (BPharm) N = 374 (53.3%)	Doctor of Pharmacy (PharmD) N = 308 (43.9%)	Master of Sciences of Pharmacy (MPharm) N = 20 (2.8%)	
**High School**	**Public School**	452 (64.4%)	278 (74.3%)	168 (54.5%)	6 (30.0%)	**<0.001**
	**Private School**	250 (35.6%)	96 (25.7%)	140 (45.5%)	14 (70.0%)	
**Academic Year**	**First**	28 (4.0%)	16 (4.3%)	12 (3.9%)	0 (0.0%)	**<0.001**
	**Second**	59 (8.4%)	37 (9.9%)	22 (7.1%)	0 (0.0%)	
	**Third**	186 (26.5%)	70 (18.7%)	116 (37.7%)	0 (0.0%)	
	**Fourth**	256 (36.5%)	158 (42.2%)	98 (31.8%)	0 (0.0%)	
	**Fifth**	134 (19.1%)	93 (24.9%)	41 (13.3%)	0 (0.0%)	
	**Sixth**	19 (2.7%)	0 (0.0%)	19 (6.2%)	0 (0.0%)	
	**Master’s**	20 (2.8%)	0 (0.0%)	0 (0.0%)	20 (100.0%)	
**Cumulative GPA** **Or Level of Achievement**	**Excellent**	156 (22.2%)	73 (19.5%)	66 (21.4%)	17 (85.0%)	**<0.001**
	**Very Good**	334 (47.6%)	178 (47.6%)	153 (49.7%)	3 (15.0%)	
	**Good**	186 (26.5%)	109 (29.1%)	77 (25.0%)	0 (0.0%)	
	**Satisfactory**	26 (3.7%)	14 (3.8%)	12 (3.9%)	0 (0.0%)	
**Work Status**	**Not Working**	451 (64.2%)	238 (63.6%)	212 (68.8%)	1 (5.0%)	**<0.001**
	**Employment**	125 (17.8%)	55 (14.7%)	51 (16.6%)	19 (95.0%)	
	**Internship or Trainee**	126 (18.0%)	81 (21.7%)	45 (14.6%)	0 (0.0%)	

^*a*^A *p-*value of less than 0.05 indicates statistical significance.

The distribution of faculty members is presented in Table 3. Most faculty members were Ph.D. degree holders (106, 61.3%), had 14.4 ± 9.3 years of work experience, clinical pharmacy & therapeutics as their exact specialty (37, 21.4%), and were full-time employees (133, 76.9%), and the most taught subjects were clinical pharmacy & therapeutics, and pharmacology & toxicology, (34, 19.7%), (33, 19.1%) respectively, while the least taught one was pharmaceutical analytical chemistry (3, 1.7%).

**Table 3:** The distribution of faculty members.

Variable		Total Faculty Members N = 173	Highest Academic Qualification			*p-*value^*a*^
			Bachelor’s Degree N = 18 (10.4%)	Master’s Degree N = 49 (28.3%)	Ph.D. N = 106 (61.3%)	
**Work Experience** **(Years)**		14.4 ± 9.3	4.5 ± 2.7	9.2 ± 4.7	18.5 ± 9.1	**<0.001**
**Country of Obtaining the Last Academic Degree** ^***b***^	**Jordan**	25 (14.5%)	3 (16.7%)	13 (26.5%)	9 (8.5%)	**<0.001**
	**United Kingdom**	26 (15.0%)	0 (0.0%)	5 (10.2%)	21 (19.8%)	
	**United States**	12 (6.9%)	0 (0.0%)	1 (2.1%)	11 (10.4%)	
	**Lebanon**	31 (17.9%)	0 (0.0%)	9 (18.4%)	22 (20.8%)	
	**Egypt**	38 (22.0%)	10 (55.6%)	8 (16.3%)	20 (18.9%)	
	**Others**	41 (23.7%)	5 (27.7%)	13 (26.5%)	23 (21.6%)	
**Exact Specialization** ^***b***^	**Clinical Pharmacy & Therapeutics**	37 (21.4%)	9 (50.0%)	12 (24.5%)	16 (15.1%)	**0.022**
	**Medicinal Chemistry & Drug Design**	26 (15.0%)	3 (16.7%)	6 (12.2%)	17 (16.0%)	
	**Microbiology & Biotechnology**	14 (8.1%)	0 (0.0%)	2 (4.1%)	12 (11.3%)	
	**Pharmacognosy & Phytochemisty**	22 (12.7%)	0 (0.0%)	5 (10.2%)	17 (16.0%)	
	**Pharmacology & Pharmacokinetics**	17 (9.8%)	1 (5.5%)	2 (4.1%)	14 (13.3%)	
	**Pharmaceutics & Industrial Pharmacy**	27 (15.6%)	2 (11.1%)	12 (24.5%)	13 (12.3%)	
	**Others**	30 (17.3%)	3 (16.7%)	10 (20.4%)	17 (16.0%)	
**Work Status**	**Full-Time Employment**	133 (76.9%)	14 (77.8%)	32 (65.3%)	87 (82.1%)	**0.070**
	**Part-Time Employment**	40 (23.1%)	4 (22.2%)	17 (34.7%)	19 (17.9%)	
**Usually Taught Courses** ^***b***^	**Pharmacology & Toxicology**	33 (19.1%)	1 (5.6%)	9 (18.4%)	23 (21.7%)	0.270
	**Clinical Pharmacy & Therapeutics**	34 (19.7%)	5 (27.8%)	11 (22.4%)	18 (17.0%)	0.478
	**Pharmaceutical Care & Pharmacy Practice**	25 (14.5%)	3 (16.7%)	8 (16.3%)	14 (13.2%)	0.842
	**Drug Design**	25 (14.5%)	2 (11.1%)	5 (10.2%)	18 (17.0%)	0.490
	**Herbal Medicine (Phytotherapy)**	19 (11.0%)	0 (0.0%)	3 (6.1%)	16 (15.1%)	0.073
	**Methods of Drug Analysis**	23 (13.3%)	4 (22.2%)	4 (8.2%)	15 (14.2%)	0.297
	**Others**	148 (7.9%)	12 (16.6%)	46 (18.4%)	90 (1.8%)	N/A

^*a*^A *p-*value of less than 0.05 indicates statistical significance.

^*b*^This question allows for multiple responses.

Knowledge among students and faculty members is presented in Table 4. A significant proportion of participants claimed not to understand the basic computational principles of AI (365, 41.7%), and there was no significant difference between students and faculty members in this regard. Regarding familiarity with AI nomenclature, the majority of participants were familiar with algorithms, machine learning, the Internet of Things (IoT), and robotics, with algorithms being the most well-known term (461, 52.7%), while familiarity with other terms such as neural networks, deep learning, and big data was relatively low. There was a statistically significant difference between students and faculty members in their familiarity with algorithms, IoT, and robotics. The participants’ understanding of AI advantages was moderate (score 4.4 ± 3 out of 10), with a statistically significant difference between students and faculty members, with students having a slightly higher score. The same was true for understanding AI disadvantages (score 3.2 ± 2.4 out of 8), AI applications (score 4.9 ± 4.3 out of 14), and its impact during COVID-19 (score 2.5 ± 2.24 out of 7); however, there was no significant difference between the two groups.

**Table 4:** Knowledge among students and faculty members.

Variable		Total N = 875	Students N = 702	Faculty Members N = 173	*p-*value^*a*^
**Understanding AI Basic Computational Principles**	**Strongly Disagree**	136 (15.5%)	103 (14.7%)	33 (19.1%)	0.069
	**Disagree**	229 (26.2%)	178 (25.4%)	51 (29.5%)	
	**Neutral**	294 (33.6%)	243 (34.6%)	51 (29.5%)	
	**Agree**	141 (16.1%)	122 (17.4%)	19 (11.0%)	
	**Strongly Agree**	75 (8.6%)	56 (8.0%)	19 (11.0%)	
**Familiar with AI Nomenclature**	**Algorithms**	461 (52.7%)	382 (54.4%)	79 (45.7%)	**0.010**
	**Machine Learning**	383 (43.8%)	321 (45.7%)	62 (35.8%)	0.057
	**Neural Networks**	180 (20.6%)	149 (21.2%)	31 (17.9%)	0.624
	**Deep Learning**	195 (22.3%)	168 (23.9%)	27 (15.6%)	0.061
	**Big Data**	226 (25.8%)	177 (25.2%)	49 (28.3%)	0.698
	**Collaborative Systems**	156 (17.8%)	128 (18.2%)	28 (16.2%)	0.574
	**Internet of Things**	404 (46.2%)	351 (50.0%)	53 (30.6%)	**<0.001**
	**Robotics**	411 (47.0%)	352 (50.1%)	59 (34.1%)	**0.001**
**Understanding AI Advantages Score out of 10**		4.4 ± 3	4.5 ± 3	4 ± 3	**0.023**
**Understanding AI Disadvantages Score out of 8**		3.2 ± 2.4	3.2 ± 2.4	2.9 ± 2.3	0.089
**Understanding AI Applications Score out of 14**		4.9 ± 4.3	5 ± 4.3	4.7 ± 4.2	0.558
**Understanding AI impact during COVID-19 Score out of 7**		2.5 ± 2.24	2.5 ± 2.27	2.4 ± 2.13	0.458
**Total Knowledge Score out of 60**		25.4 ± 13.1	25.9 ± 12.9	23.3 ± 13.8	**0.023**

^*a*^A *p-*value of less than 0.05 indicates statistical significance.

Attitudes towards AI in pharmacy and pharmacy practice are presented in Table 5. The majority of the participants agreed that AI will improve and revolutionize clinical pharmacy practice (593, 67.8%) and other general pharmacy sciences (624, 71.3%). However, some participants disagreed or had neutral attitudes towards the impact of AI on healthcare professionals. Interestingly, participants had varying opinions about the impact of AI on the pharmacy profession. While some believed that AI would reduce the number of general pharmacists needed (438, 50.1%), others believed that it would increase the number of specialized pharmacists needed (402, 45.9%). Nonetheless, a significant percentage of participants agreed that AI will never make healthcare professionals expendable (471, 53.8%). It is important to note that attitudes towards AI were not uniform among the participants. Some saw AI as a partner that will help them perform their duties effectively (515, 58.9%), while others viewed it as a competitor that will take over their jobs (319, 36.4%). However, it was generally agreed that pharmacy students should receive teaching in AI during their study (629, 60.5%), and teaching in AI will be beneficial for their career (550, 62.9%). When asked to indicate the specialty most likely to be impacted by AI in the near future, the highest percentage of responses answered pharmaceutical statistics (472, 53.9%), followed by drug design (435, 49.7%). While the two fields with the lowest percentage of respondents were herbal medicine (115, 13.1%) and pathophysiology (138, 15.8%).

**Table 5:** Attitudes towards AI in pharmacy and pharmacy practice.

Variable		Total N = 875
**AI will improve and revolutionize clinical pharmacy practice**	**Strongly Disagree**	4 (0.5%)
	**Disagree**	42 (4.8%)
	**Neutral**	263 (27.0%)
	**Agree**	391 (44.7%)
	**Strongly Agree**	202 (23.1%)
**AI will improve and revolutionize other general pharmacy sciences**	**Strongly Disagree**	2 (0.2%)
	**Disagree**	39 (4.5%)
	**Neutral**	210 (24.0%)
	**Agree**	436 (49.8%)
	**Strongly Agree**	188 (21.5%)
**Most of the non-specialized healthcare providers will be replaced by foreseeable future**	**Strongly Disagree**	47 (5.4%)
	**Disagree**	202 (23.1%)
	**Neutral**	280 (32.0%)
	**Agree**	250 (28.6%)
	**Strongly Agree**	96 (11.0%)
**Most General Physicians will be replaced by foreseeable future**	**Strongly Disagree**	60 (6.9%)
	**Disagree**	239 (27.3%)
	**Neutral**	281 (32.1%)
	**Agree**	204 (23.3%)
	**Strongly Agree**	91 (10.4%)
**The impact of AI alone will reduce the number of general pharmacists (non-specialist) that are needed**	**Strongly Disagree**	33 (3.8%)
	**Disagree**	142 (16.2%)
	**Neutral**	262 (29.9%)
	**Agree**	300 (34.3%)
	**Strongly Agree**	138 (15.8%)
**The impact of AI will increase the number of specialized pharmacists that are needed**	**Strongly Disagree**	34 (3.9%)
	**Disagree**	128 (14.6%)
	**Neutral**	311 (35.5%)
	**Agree**	268 (30.6%)
	**Strongly Agree**	134 (15.3%)
**AI will never make Healthcare professionals expendable**	**Strongly Disagree**	23 (2.6%)
	**Disagree**	121 (13.8%)
	**Neutral**	260 (29.7%)
	**Agree**	306 (35.0%)
	**Strongly Agree**	165 (18.9%)
**I do not expect AI to have wide use in pharmaceutical practice in the future**	**Strongly Disagree**	65 (7.4%)
	**Disagree**	221 (25.3%)
	**Neutral**	317 (36.2%)
	**Agree**	185 (21.1%)
	**Strongly Agree**	87 (9.9%)
**I predict and expect that AI will have a prosperous future in pharmaceutical practice**	**Strongly Disagree**	18 (2.1%)
	**Disagree**	75 (8.6%)
	**Neutral**	267 (30.5%)
	**Agree**	350 (40.0%)
	**Strongly Agree**	165 (18.9%)
**I am less likely to consider a position in different pharmacy careers, given the advancement of AI**	**Strongly Disagree**	56 (6.4%)
	**Disagree**	203 (23.2%)
	**Neutral**	297 (33.9%)
	**Agree**	205 (23.4%)
	**Strongly Agree**	114 (13.0%)
**All pharmacy students should receive teaching in AI during their study**	**Strongly Disagree**	36 (4.1%)
	**Disagree**	76 (8.7%)
	**Neutral**	234 (26.7%)
	**Agree**	303 (23.2%)
	**Strongly Agree**	326 (37.3%)
**Teaching in AI will be beneficial for my career**	**Strongly Disagree**	32 (3.7%)
	**Disagree**	69 (7.9%)
	**Neutral**	224 (25.6%)
	**Agree**	224 (25.6%)
	**Strongly Agree**	326 (37.3%)
**At the end of my pharmacy degree, I will be familiar in using basic healthcare AI tools for pharmaceutical care & practice if required**	**Strongly Disagree**	74 (8.5%)
	**Disagree**	108 (12.3%)
	**Neutral**	280 (32.0%)
	**Agree**	198 (22.6%)
	**Strongly Agree**	215 (24.6%)
**At the end of my pharmacy degree, I will have a better understanding of the methods used to assess healthcare AI algorithm performance**	**Strongly Disagree**	89 (10.2%)
	**Disagree**	133 (15.2%)
	**Neutral**	295 (33.7%)
	**Agree**	178 (20.3%)
	**Strongly Agree**	180 (20.6%)
**Overall, at the end of my pharmacy degree, I feel I will possess the knowledge needed to work with AI in routine clinical practice**	**Strongly Disagree**	93 (10.6%)
	**Disagree**	139 (15.9%)
	**Neutral**	292 (33.4%)
	**Agree**	171 (19.5%)
	**Strongly Agree**	180 (20.6%)
**Medical and pharmaceutical AI are expected to be used more often in the future**	**Strongly Disagree**	34 (3.9%)
	**Disagree**	76 (8.7%)
	**Neutral**	236 (27.0%)
	**Agree**	262 (29.9%)
	**Strongly Agree**	267 (30.5%)
**In pharmacy practice, the usage of AI would contribute in providing optimal pharmaceutical care and improving patients health outcomes**	**Strongly Disagree**	28 (3.2%)
	**Disagree**	96 (11.0%)
	**Neutral**	282 (32.2%)
	**Agree**	256 (29.3%)
	**Strongly Agree**	213 (24.3%)
**In general, most AI technology systems provide accurate and trusted health-related information**	**Strongly Disagree**	24 (2.7%)
	**Disagree**	104 (11.9%)
	**Neutral**	309 (35.3%)
	**Agree**	248 (28.3%)
	**Strongly Agree**	190 (21.7%)
**Total Attitude Score out of 90**		62.5 ± 10.7

Perceptions of students and faculty members on the impact of AI on various pharmacy specialties are presented in Table 6. There is some variation in perceptions of the impact of AI on different pharmacy specialties, but in general, a majority of respondents perceive AI as having an impact on most pharmacy specialties. Both students and academics believed that Pharmaceutical Statistics (472, 53.9%), Drug Design (435, 49.7%), and Pharmaceutical Marketing and Promotion (388, 44.3%) were the most frequent courses that would be positively affected by AI.

**Table 6:** Perceptions of students and faculty members on the impact of AI on various pharmacy specialties.

Variable	Total N = 875	Students N = 702	Faculty Members N = 173	*p-*value^*a*^
**Pharmaceutical Statistics**	472 (53.9%)	377 (53.7%)	95 (54.9%)	0.775
**Pharmacoeconomics**	351 (40.1%)	281 (40.0%)	70 (40.5%)	0.917
**Pathophysiology**	138 (15.8%)	117 (16.7%)	21 (12.1%)	0.143
**Pharmacology**	281 (32.1%)	233 (33.2%)	48 (27.7%)	0.169
**Biopharmaceutics and Pharmacokinetics**	311 (35.5%)	240 (34.2%)	71 (41.0%)	0.092
**Pharmacogenomics**	369 (42.2%)	296 (42.2%)	73 (42.2%)	0.994
**Biotechnology and Biomedicine**	347 (39.7%)	291 (41.5%)	56 (32.4%)	**0.029**
**Pharmaceutical Marketing and Promotion**	388 (44.3%)	315 (44.9%)	73 (42.2%)	0.526
**Industrial Pharmacy and Drug Delivery**	352 (40.2%)	290 (41.3%)	62 (35.8%)	0.189
**Clinical Pharmacy and Therapeutics**	263 (30.1%)	205 (29.2%)	58 (33.5%)	0.267
**Pharmaceutical Care and Pharmacy Practice**	239 (27.3%)	186 (26.5%)	53 (30.6%)	0.274
**Pharmaceutical Quality and Regulatory Affairs**	284 (32.5%)	242 (34.5%)	42 (24.3%)	**0.010**
**Drug Design**	435 (49.7%)	346 (49.3%)	89 (51.4%)	0.611
**Herbal Medicine (Phytotherapy)**	115 (13.1%)	95 (13.5%)	20 (11.6%)	0.492
**Methods of Drug Analysis**	331 (37.8%)	267 (38.0%)	64 (37.0%)	0.801

^*a*^A *p-*value of less than 0.05 indicates statistical significance.

The most frequently used references of information related to pharmacy practice are presented in Table 7. The most frequently used reference for information was the internet (i.e., Google^®^ search), reported by 586 (67.0%) of the total participants, followed by databases and applications, used by 429 (49.0%), while interactive learning platforms were the least frequently used reference, used by 170 (19.4%), with no significant difference between students and faculty members. Other frequently used references shown in the table included evidence-based resources (407, 46.5%), books (384, 43.9%), healthcare providers (340, 38.9%), drug information leaflets (333, 38.1%), scientific journals and articles (290, 33.1%), training guide manuals (254, 29.0%), and social media platforms (219, 25.0%). When comparing the responses of both groups, significant differences were found. Students reported using training guide manuals, drug information leaflets, healthcare providers, and social media platforms more frequently than faculty members did, while faculty members reported using evidence-based resources as well as scientific journals and articles more frequently than students did.

**Table 7:** Most frequently used reference of information related to pharmacy practice.

Variable	Total N = 875	Students N = 702	Faculty Members N = 173	*p-*value^*a*^
**Internet (Google**^**®**^ **Search, …)**	586 (67.0%)	479 (68.2%)	107 (61.8%)	0.110
**Training-Guide Manuals**	254 (29.0%)	221 (31.5%)	33 (19.1%)	**0.001**
**Drug Information Leaflets**	333 (38.1%)	282 (40.2%)	51 (29.5%)	**0.009**
**Healthcare Providers (Pharmacists, Physicians, Nurses, …)**	340 (38.9%)	294 (41.9%)	46 (26.6%)	**<0.001**
**Databases & Applications (Lexicomp** ^**®**^ **, Drugs.com** ^**®**^ **, Micromedex** ^**®**^ **, Medscape** ^**®**^ **, …)**	429 (49.0%)	335 (47.7%)	94 (54.3%)	0.119
**Books (BNF** ^**®**^ **, DIH** ^**®**^ **, Pharmacology Textbooks, …)**	384 (43.9%)	306 (43.6%)	78 (45.1%)	0.722
**Evidence-based Resources (Guidelines, DiPiro Pharmacotherapy** ^**®**^ **, UpToDate** ^**®**^ **, …)**	407 (46.5%)	313 (44.6%)	94 (54.3%)	**0.021**
**Social Media Platforms (Facebook** ^**®**^ **, YouTube** ^**®**^ **, Instagram** ^**®**^ **, …)**	219 (25.0%)	189 (26.9%)	30 (17.3%)	**0.009**
**Interactive Learning Platforms (Coursera** ^**®**^ **, edX** ^**®**^ **, Udemy** ^**®**^ **, …)**	170 (19.4%)	140 (19.9%)	30 (17.3%)	0.438
**Scientific Journals and Articles**	290 (33.1%)	198 (28.2%)	92 (53.2%)	**<0.001**

^*a*^A *p-*value of less than 0.05 indicates statistical significance.

Exposure to AI or AI sources of information is presented in Table 8. It shows that a significant proportion of participants had not been exposed to AI and their sources of information (374, 42.7%). Also, it shows that (175, 20.0%) reported that they worked on clinical research involving AI, while scientific conferences and social media platforms were reported by (208, 23.8%) and (258, 29.5%) of the participants, respectively. Courses on AI/Machine Learning were taken by (134, 15.3%) of the participants, and (104, 11.9%) worked on computer science projects involving AI. Friends or family in the medical field were reported by (182, 20.8%) of the participants, while (133, 15.2%) relied on friends or family in non-medical fields. The exposure to AI by medical or pharmacy staff at training sites was reported by (140, 16.0%) of the participants. Lastly, only (102, 11.7%) reported exposure to AI as part of the pharmacy school curriculum. Statistically significant differences were observed between faculty members and students in exposure to AI through clinical research, scientific conferences, and social media platforms, as well as exposure to AI through medical or pharmacy staff at training sites.

**Table 8:** Exposure to AI and its sources of information.

Variable	Total N = 875	Students N = 702	Faculty Members N = 173	*p-*value^*a*^
**Haven’t exposed to AI**	374 (42.7%)	310 (44.2%)	64 (37.0%)	0.088
**Courses on AI / machine learning**	134 (15.3%)	112 (16.0%)	22 (12.7%)	0.290
**Computer science projects involving AI**	104 (11.9%)	86 (12.3%)	18 (10.4%)	0.502
**Clinical research involving AI**	175 (20.0%)	129 (18.4%)	46 (26.6%)	**0.016**
**Scientific Conferences**	208 (23.8%)	155 (22.1%)	53 (30.6%)	**0.018**
**Social Media Platforms (e.g., Facebook**^**®**^**, Twitter**^**®**^**, LinkedIn**^**®**^**, ResearchGate**^**®**^ **…etc.)**	258 (29.5%)	218 (31.1%)	40 (23.1%)	**0.040**
**Friends or Family in Medical Field**	182 (20.8%)	148 (21.1%)	34 (19.7%)	0.678
**Friends or Family in Non-medical Field**	133 (15.2%)	108 (15.4%)	25 (14.5%)	0.759
**Medical or Pharmacy Staff at Training Sites**	140 (16.0%)	125 (17.8%)	15 (8.7%)	**0.003**
**Part of the Pharmacy School Education Curriculum**	102 (11.7%)	87 (12.4%)	15 (8.7%)	0.172

^*a*^A *p-*value of less than 0.05 indicates statistical significance.

Practices towards AI in pharmacy practice are presented in Tables 9 and 10. The participants were asked if they would consider using a clinical workflow where patients’ diagnostic information undergoes AI analysis and is subsequently reviewed by a specialized pharmacist. The results showed that (489, 55.9%) of the total participants would consider using this workflow. The difference in responses between the groups was not statistically significant. However, a significant proportion of participants were not sure if they would consider using this workflow (264, 30.2%). Regarding the currently applied AI in practice among pharmacy students and faculty members, (153, 17.5%) of the respondents attended any AI seminar in the last year, and (110, 12.6%) attended a workshop about AI in healthcare systems. Additionally, (318, 36.3%) of the respondents had read an article on AI in pharmacy, and only (70, 8.0%) had received a verified certificate in AI. The majority of respondents, around (556, 63.6%), expressed their willingness to contribute to adding educational material related to AI to the curriculum of the College of Pharmacy. Moreover, the majority of respondents, around (562, 64.2%), reported they would follow up on the latest updates related to AI in healthcare, with (209, 23.9%) of them always following up. The *p-*values indicate that the differences between the two groups of respondents are not statistically significant, except for attending any AI seminar in the last year, where the faculty members had a slightly higher percentage of attendance.

**Table 9:** Practice of students and faculty members of AI in pharmacy practice.

Variable		Total N = 875	Students N = 702	Faculty Members N = 173	*p-*value^*a*^
**In the future, would you consider using the following clinical workflow as a pharmacist? Patients’ diagnostic information undergo artificial intelligence analysis. The specialized pharmacist subsequently reviews both the information and the artificial intelligence findings**	**Yes**	489 (55.9%)	393 (56.0%)	96 (55.5%)	0.437
	**No**	122 (13.9%)	93 (13.2%)	29 (16.8%)	
	**Not Sure**	264 (30.2%)	216 (30.8%)	48 (27.7%)	

^*a*^A *p-*value of less than 0.05 indicates statistical significance.

**Table 10:** Current applied AI practice among pharmacy students and faculty members.

Variable		Total N = 875	Students N = 702	Faculty Members N = 173	*p-*value^*a*^
**Attended any AI seminar the last year**	**Yes**	153 (17.5%)	112 (16.0%)	41 (23.7%)	**0.016**
	**No**	722 (82.5%)	590 (84.0%)	132 (76.3%)	
**Attended a workshop about AI in healthcare systems**	**Yes**	110 (12.6%)	83 (11.8%)	27 (15.6%)	0.179
	**No**	765 (87.4%)	619 (88.2%)	146 (84.4%)	
**Read any article on AI in pharmacy**	**Yes**	318 (36.3%)	246 (35.0%)	72 (41.6%)	0.107
	**No**	557 (63.7%)	456 (65.0%)	101 (58.4%)	
**Received a verified certificate in AI**	**Yes**	70 (8.0%)	58 (8.3%)	12 (6.9%)	0.565
	**No**	805 (92.0%)	644 (91.7%)	161 (93.1%)	
**After completing this survey, will you contribute to adding educational material related to AI in the curriculum of the College of Pharmacy**	**Strongly Disagree**	25 (2.9%)	23 (3.3%)	2 (1.2%)	0.614
	**Disagree**	51 (5.8%)	42 (6.0%)	9 (5.2%)	
	**Neutral**	243 (27.8%)	193 (27.5%)	50 (28.9%)	
	**Agree**	313 (35.8%)	252 (35.9%)	61 (35.3%)	
	**Strongly Agree**	243 (27.8%)	192 (27.4%)	51 (29.5%)	
**After completing this survey, will you follow up on the latest updates related to AI in healthcare**	**Always**	209 (23.9%)	162 (23.1%)	47 (27.2%)	0.344
	**Often**	353 (40.3%)	281 (40.0%)	72 (41.6%)	
	**Sometimes**	242 (27.7%)	198 (28.2%)	44 (25.4%)	
	**Never**	42 (4.8%)	34 (4.8%)	8 (4.6%)	
	**Do Not Apply**	29 (3.3%)	27 (3.8%)	2 (1.2%)	
**Total Practice Score out of 231**		163.6 ± 32.6	163.6 ± 33.4	163.6 ± 29.4	0.979

^a^A *p-*value of less than 0.05 indicates statistical significance.

The parameters affecting the KAP score among the study participants are presented in Table 11. The analysis showed that country of residence had a significant effect on the mean total knowledge score, with Jordanian students having the highest score (47.4 ± 22.3) compared to other countries (*p* < 0.001). Moreover, the academic path also had a significant effect, where students had a higher mean total knowledge score than faculty members (43.1 ± 21.4 vs. 38.9 ± 23, *p* = 0.023). However, gender, citizenship, marital status, and work place did not show any significant effect on the knowledge score. The monthly household income had a significant effect on the knowledge score, where those from the upper class had a higher mean total knowledge score (45.3 ± 21.6) than those from the lower and middle classes (*p =* 0.001). In addition, the type of university also had a significant effect on the knowledge score, where private university students had a higher mean total knowledge score (45 ± 20.8) compared to governmental university students (*p =* 0.001). Moreover, tech-savviness had a significant effect on the knowledge score, where those who strongly agreed with being tech-savvy had the highest mean total knowledge score (54 ± 21.7) compared to other groups (*p <* 0.001). Furthermore, the academic year and cumulative GPA or level of achievement had a significant effect on the knowledge score. Fourth- and fifth-year students had a higher mean total knowledge score compared to first-year students (*p <* 0.001), and those with an excellent level of achievement had a higher mean total knowledge score than those with a satisfactory level (*p =* 0.004). However, other variables did not show any significant effect on the knowledge score. Overall, the results suggest that several socio-demographic and educational factors may influence the knowledge score among pharmacy students and faculty members. Regarding the factors that affect the attitude score of pharmacy students and faculty members, the monthly household income, university type, and tech-savvy are statistically significant factors affecting the attitude score, with *p-*values < 0.05. Specifically, the mean attitude score was significantly higher in upper class income and private universities than in lower class income and government universities. The mean attitude score was also significantly higher for those who strongly agreed with their tech-savviness compared to other groups. Other independent variables of students’ characteristics, such as high school, academic year, level of achievement, and work status, also showed statistically significant differences in attitude score with a *p-*value (0.043, 0.047, < 0.001, and 0.042, respectively). The various factors that affected the mean total practice score of pharmacy students and faculty members were gender, university type, and level of tech-savviness, significantly impacted the practice score. Females and participants from private universities had a higher practice score than males and those from government universities. Strongly agreeing with being tech-savvy also significantly impacted the practice score. Among pharmacy students, academic year and cumulative GPA were significant factors that affected the practice score. Fourth- and fifth-year students had a higher practice score than first-year students. Students with an excellent level of achievement had a significantly higher practice score than those with a satisfactory level of achievement.

**Table 11:** Parameters affecting the KAP scores.

Variable			Knowledge		Attitude		Practice	
			Mean Total Score (%) ± SD	*p-*value^*a*^	Mean Total Score (%) ± SD	*p-*value^*a*^	Mean Total Score (%) ± SD	*p-*value^*a*^
**Independent**	**Country**	**Jordan**	47.4 ± 22.3	**<0.001**	69.1 ± 10.1	**<0.001**	72.2 ± 13	**<0.001**
		**Egypt**	38.7 ± 21.8		66.4 ± 10.8		68.6 ± 12.8	
		**Lebanon**	35 ± 19.9		71.7 ± 15.8		67.5 ± 17.1	
		**Libya**	42.1 ± 19.8		71.5 ± 12.4		75.1 ± 13.8	
		**Palestine**	44.1 ± 21.3		68.3 ± 8.4		69.2 ± 12.3	
		**Saudi Arabia**	53.4 ± 25.5		72.4 ± 9.7		68.2 ± 12.3	
	**Academic Path**	**Students**	43.1 ± 21.4	**0.023**	69.5 ± 11.9	0.960	70.8 ± 14.5	0.979
		**Faculty Members**	38.9 ± 23		69.4 ± 12		70.8 ± 12.7	
	**Age (Years)**		25.8 ± 9.4	0.273	25.8 ± 9.4	0.129	25.8 ± 9.4	0.275
	**Gender**	**Males**	41.7 ± 22.5	0.616	70 ± 13	0.433	69.2 ± 15.6	**0.034**
		**Females**	42.5 ± 22.5		69.3 ± 11.4		71.5 ± 13.4	
	**Citizenship**	**Local**	42 ± 21.8	0.348	69.6 ± 12.2	0.373#	70.7 ± 14.2	0.336
		**International**	44.3 ± 21.6		68.7 ± 9.1		72.1 ± 13.5	
	**Material Status**	**Single**	43.3 ± 21.5	0.058	69.3 ± 11.7	0.355	70.8 ± 14.3	0.316
		**Married**	39.9 ± 22.6		70.2 ± 12		71.9 ± 12.6	
	**Monthly Household Income**	**Lower Class**	38.6 ± 21.3	**0.001**	67 ± 10.6	**<0.001**	69.6 ± 14.3	0.120
		**Middle Class**	39 ± 21.9		69.1 ± 12		70.4 ± 14.7	
		**Upper Class**	45.3 ± 21.6		71.4 ± 12.3		72 ± 13.4	
	**University**	**Governmental**	40.2 ± 22.3	**0.001**	67.9 ± 11.4	**<0.001**	69.1 ± 13.9	**<0.001**
		**Private**	45 ± 20.8		71.5 ± 12.2		73.1 ± 14.1	
	**Work Place**	**Pharmacy**	42 ± 19.6	0.286	69.8 ± 12.6	0.083	71.6 ± 14.6	0.749
		**University**	38.9 ± 23		69.4 ± 12		70.8 ± 12.7	
		**Others**	43.6 ± 21.6		74.1 ± 15.2		72.3 ± 13.7	
	**Tech-savvy**	**Strongly Disagree**	25.6 ± 16.8	**<0.001**	62.3 ± 12.6	**<0.001**	61.9 ± 13.6	**<0.001**
		**Disagree**	30.7 ± 18.9		66.4 ± 10.5		68.1 ± 15.3	
		**Neutral**	40.8 ± 20.2		67.6 ± 10.7		69.4 ± 13.3	
		**Agree**	48.1 ± 20.6		71.5 ± 11.1		73.2 ± 13.8	
		**Strongly Agree**	54 ± 21.7		76.4 ± 13.1		76.4 ± 12.8	
	**Students**							
	**Current Major**	**BPharm**	41.6 ± 22	0.091	68.7 ± 11.2	0.301	69.9 ± 14	0.165
		**PharmD**	44.4 ± 20.8		69.7 ± 12.1		71.4 ± 14.9	
	**High School**	**Public School**	42 ± 22.1	0.052	68.8 ± 11.5	**0.043**	70.2 ± 14	0.125
		**Private School**	45.2 ± 20		70.7 ± 12.5		72 ± 15.3	
	**Academic Year**	**First**	32.2 ± 25.1	**<0.001**	66.5 ± 12.6	**0.047**	62.9 ± 20.6	**0.014**
		**Second**	39.4 ± 21.2		71 ± 14.1		68.3 ± 15	
		**Third**	39.4 ± 20.7		69.8 ± 11.7		72.4 ± 15	
		**Fourth**	45.3 ± 20.2		67.8 ± 10.9		70.9 ± 12.8	
		**Fifth**	46.5 ± 23		70.7 ± 11.6		70.9 ± 14.3	
	**Cumulative GPA** **Or Level of Achievement**	**Excellent**	46.5 ± 21.9	**0.004**	72.6 ± 13	**<0.001**	74.3 ± 15	**<0.001**
		**Very Good**	43.4 ± 21.3		69.3 ± 11		70 ± 14.1	
		**Good**	41.4 ± 21.1		68.2 ± 12.1		71 ± 13.4	
		**Satisfactory**	31 ± 18.3		62.5 ± 10.4		60 ± 17	
	**Work Status**	**Not Working**	42.5 ± 22.2	0.515	69 ± 11.6	**0.042**	70.3 ± 14.9	0.073
		**Employment**	43.6 ± 20.8		71.9 ± 14.4		73.5 ± 13.3	
		**Trainee**	44.9 ± 19.3		68.7 ± 10		70.1 ± 13.7	
	**Faculty Members**							
	**Highest Academic Qualification**	**Bachelor’s**	38.9 ± 29.1	0.697	66.7 ± 7.7	0.456	72.5 ± 11.2	0.808
		**Master’s**	41.2 ± 22.4		68.8 ± 13.1		70.2 ± 11.3	
		**Ph.D.**	37.8 ± 22.3		70.2 ± 12		70.8 ± 13.6	
	**Country of Obtaining the Last Academic Degree**	**Jordan**	40 ± 23.1	**0.002**	66.1 ± 10.8	**0.003**	69 ± 12.1	0.348
		**United Kingdom**	41.4 ± 22.3		67.9 ± 9.1		71.1 ± 12.1	
		**United States**	60.3 ± 23.2		78.1 ± 11.8		76.9 ± 11.9	
		**Lebanon**	30.5 ± 16.7		72 ± 13.2		71.2 ± 12.6	
		**Egypt**	33.1 ± 25		65 ± 9.3		68 ± 12.3	
		**Others**	42.2 ± 21.7		72.1 ± 13.5		72.2 ± 14	
	**Exact Specialization**	**Clinical Pharmacy & Therapeutics**	45 ± 23.6	0.6	68.1 ± 13.2	0.728	71.7 ± 12.6	0.656
		**Medicinal Chemistry & Drug Design**	33.7 ± 24.7		70.8 ± 12.9		74.2 ± 12.8	
		**Microbiology & Biotechnology**	34.5 ± 20.5		73.2 ± 12.3		72 ± 15.7	
		**Pharmacognosy & Phytochemisty**	37.2 ± 22.7		70.8 ± 10.5		70.5 ± 13	
		**Pharmacology & Pharmacokinetics**	39.8 ± 24.1		70.8 ± 10.4		69.2 ± 9.2	
		**Pharmaceutics & Industrial Pharmacy**	39.1 ± 21.6		68.2 ± 8.3		70.3 ± 10.7	
		**Others**	38.6 ± 23.2		67.6 ± 14.2		67.7 ± 14.7	
	**Work Status**	**Full-Time**	38.6 ± 23.2	0.732	69 ± 11	0.488	70.6 ± 13.1	0.676
		**Part-Time**	40 ± 22.5		70.8 ± 14.8		71.5 ± 11.8	
	**Work Experience (Years)**		14.4 ± 9.3	0.636	14.4 ± 9.3	0.088	14.4 ± 9.3	0.424

^*a*^A *p-*value of less than 0.05 indicates statistical significance, calculated by independent *t-*test or ANOVA or Pearson’s *r* when appropriate.

A comparison of KAP scores between pharmacy students and faculty members is presented in Tables 12 and 13. The results show that the mean score for knowledge was 42.3 ± 21.8 for all participants, with students having a slightly higher mean score of 43.1 ± 21.4 compared to faculty members’ mean score of 38.9 ± 23 (*p* = 0.023). However, there was no significant difference in attitude or practice scores between students and faculty members (*p* = 0.960 and *p* = 0.979, respectively). A correlation analysis was conducted to examine the relationships between the variables. The results suggest that there is a significant positive relationship between participants’ scores on the KAP survey and their scores on the knowledge, attitude, and practice subscales. Additionally, there are moderate-to-strong positive correlations between the three subscales, indicating that participants who score highly on one subscale are likely to score highly on the others as well.

**Table 12:** Comparison of KAP scores between pharmacy students and faculty members.

Variable	Total N = 875	Students N = 702	Faculty Members N = 173	*p-*value^*a*^
**Knowledge Score (100%)**	42.3 ± 21.8	43.1 ± 21.4	38.9 ± 23	**0.023**
**Attitude Score (100%)**	69.5 ± 11.9	69.5 ± 11.9	69.4 ± 12	0.960
**Practice Score (100%)**	70.8 ± 14.1	70.8 ± 14.5	70.8 ± 12.7	0.979
**KAP Score (100%)**	60.9 ± 12.2	61.1 ± 12.2	59.7 ± 12.2	0.168

^*a*^A *p-*value of less than 0.05 indicates statistical significance.

**Table 13:** Correlation analysis of KAP scores between pharmacy students and faculty members.

Variable	Total N = 875	Pearson Correlation Coefficient (*r*)	*p-*value^*a*^
**Total Knowledge Score (100%)**	42.3 ± 21.8	**0.824**	**<0.001**
**Total Attitude Score (100%)**	69.5 ± 11.9	**0.697**	**<0.001**
**Total Practice Score (100%)**	70.8 ± 14.1	**0.738**	**<0.001**
**Total KAP Score (100%)**	60.9 ± 12.2	**1**	**<0.001**

^*a*^A *p-*value of less than 0.05 indicates statistical significance, calculated by Pearson’s *r*.

## Discussion

The term “artificial intelligence” (AI) refers to the branch of computer science that focuses on creating computer programs that can carry out tasks that would normally require human intellect [33]. The use of this technology might significantly alter clinical pharmacy practice. Learning to use these technologies in a way that reveals novel health data trends and really benefits patients is one of the challenges for clinical pharmacy practice [34, 35]. We carried out this study to investigate the knowledge, attitudes, and practices (KAP) of 875 pharmacy students and faculty members regarding AI in Jordan, Palestine, Lebanon, Egypt, Saudi Arabia, and Libya, with a particular focus on identifying the predictive factors behind their understanding of the principles and practical applications of AI in healthcare processes.

The demographic characteristics of the respondents were similar to those of the general population in the MENA region [36], as the majority were young, native local citizens. However, 70.3% of the respondents were female, which reflects the dominance of female gender in the pharmaceutical sector and is consistent with previous research [19]. A large proportion of the respondents self-rated themselves as being tech-savvy and having no problems dealing with technology. These findings are also comparable with those of previous studies [37, 38].

Regarding the KAP analysis, our results showed that the overall level of knowledge about AI among the study participants was moderate, with the mean knowledge score being 42.3 ± 21.8 out of 100. These findings suggest that both groups have some understanding of the principles of AI, with students having a significantly higher knowledge score than faculty members, which may reflect the fact that students have more recently been exposed to AI than faculty members, but there is a significant gap in their practical application of this technology in healthcare processes. This discrepancy may be attributed to the generational difference in exposure to AI technologies. Students, being digital natives, are more likely to encounter AI tools during their education. Moreover, there is a need for incorporating AI-related courses into pharmacy curricula and continuing education and training programs to improve their knowledge, skills, and practical application of AI in pharmacy practice, especially for pharmacy faculty members, in order to keep pace with the rapidly evolving field. Comparably, a study carried out in Saudi Arabia reported that pharmacy students showed good awareness of AI. Moreover, they reported that the majority of the students had positive perceptions about the concepts, benefits, and implementation of AI [26].

Moreover, participants had a greater understanding of AI nomenclature than AI advantages, indicating that they might be more familiar with the terms and concepts associated with AI than with its potential benefits and drawbacks. This gap could be due to the lack of formal education and training in AI for pharmacy students and faculty members, which is consistent with previous research [39, 40]. As a result, the findings highlight the need for incorporating more education and training on AI-related courses in pharmacy curricula to improve students’ knowledge and skills in this area, which could lead to more practical application of AI in pharmacy practice, with particular attention to basic computational principles and AI nomenclature.

We also found that the attitude towards AI among pharmacy students and faculty members is positive, but there are still concerns about the impact of AI on job security and patient safety. These concerns should be addressed through open communication, education, and collaboration among pharmacists, healthcare professionals, and AI technology experts. In a systematic review examining healthcare students’ attitudes, knowledge, and skills in AI, it was reported that 76% of healthcare students had a positive and promising attitude towards AI in the clinical profession and its use in the future; however, 24% of the students considered AI a threat to healthcare fields and had a negative attitude towards it [20]. These findings resonate with a broader shift in healthcare, where AI is increasingly seen as a valuable partner in clinical decision-making and patient care [4].

The present study also revealed that pharmacy students and faculty members had limited experience using AI tools in their practice, which suggests a need for incorporating AI education and training into pharmacy curricula. This finding is consistent with previous studies that reported a gap between the potential of AI in healthcare and the actual implementation of AI in clinical practice due to a lack of knowledge and skills among healthcare providers [41]. Overall, this suggests that AI is expected to be used more often in medical and pharmaceutical practice in the future. Therefore, it is imperative for pharmacy students to acquire the knowledge and skills needed to work with AI in routine clinical practice.

Our study results are consistent with previous research on digital health adoption in pharmacy education. For example, a recent study conducted by the International Pharmaceutical Federation (FIP) around the world found that a large proportion (57%) of pharmacy schools do not offer any digital health education, similar to our finding of low levels of AI adoption in pharmacy education [42]. Open dialogues among pharmacists, healthcare professionals, and AI experts are essential to establish ethical guidelines and ensure responsible AI integration into pharmacy practice [43, 44].

In addition, we identified several predictors of KAP toward using AI in pharmacy practice, such as high income, a strong educational level and background, and previous experience with technologies, which can inform the development of targeted continuing education and training programs to keep up with the rapid development of technologies and their applications in pharmacy and to address the specific needs of different groups of pharmacists.

Interestingly, our results showed a positive correlation between knowledge about AI and attitudes towards AI. This indicates that enhancing knowledge and awareness of AI among pharmacy students and faculty members may lead to increased acceptance and adoption of AI tools in pharmacy practice. Moreover, our results revealed a significant positive correlation between AI knowledge and overall KAP scores, which suggests that knowledge is a key determinant of behavior change towards the adoption of AI in pharmacy practice.

Overall, we suggest that AI can be viewed as a potential partner for pharmacists in improving the quality of patient care and advancing pharmacy practice. However, it is important to continue monitoring and studying the impact of AI on pharmacy practice and job security in order to address any potential concerns and ensure that the integration of AI into pharmacy practice is done in a responsible and ethical manner. Future research should focus on identifying the barriers and facilitators to the implementation of AI technology in pharmacy practice and on developing and evaluating educational interventions aimed at improving the practical skills of pharmacists in this area.

The practical implications of our study are far-reaching. Firstly, it emphasizes the importance of AI education in pharmacy curricula. References to AI literature, computational principles, and AI nomenclature should be integrated into coursework. Additionally, practical workshops and experiential learning opportunities can bridge the gap between knowledge and practice. Secondly, our findings underscore the significance of addressing concerns related to job security and patient safety associated with AI adoption. Clear guidelines and ethical frameworks for AI utilization in pharmacy practice are essential. Lastly, future research should focus on exploring AI applications across various pharmacy domains, offering innovative solutions to healthcare challenges. This can include AI-driven drug discovery, personalized medication regimens, and real-time medication adherence monitoring.

This study has some limitations. Firstly, we focused only on some countries in the MENA region, which may limit the generalizability of our findings to other regions or settings. Secondly, our study relied on self-reported data, which may be subject to response bias. Finally, our study focused only on pharmacy students and faculty members and did not include other healthcare providers, which may limit the generalizability of our findings to the broader healthcare context. The cross-sectional design limits our ability to establish causal relationships or track changes in knowledge, attitudes, and practices over time. Moreover, while efforts were made to design culturally sensitive survey instruments, variations in language and cultural nuances could influence participants’ interpretation of questions. Finally, the depth of participants’ AI understanding and the potential ethical and regulatory implications of AI integration warrant further exploration. Future studies could overcome these limitations by using a longitudinal design to investigate the effects of AI-related education on students’ knowledge and attitudes and by expanding the sample to include other regions and countries.

## Conclusion

The study provides valuable insights into the current state of KAP among pharmacy students and faculty members towards AI in pharmacy practice. Although there is moderate knowledge and positive attitudes towards AI in pharmacy practice, there is still room for improvement in integrating AI education into pharmacy curricula and practice. The study underscores the importance of continuous professional development in AI for both students and faculty members to ensure their readiness for the evolving healthcare landscape. These insights provide a basis for targeted educational interventions aiming to reinforce AI-related competencies among pharmacy professionals. By addressing the identified gaps, we can better equip future pharmacists to effectively harness AI’s potential in healthcare. It is recommended that pharmacy practitioners proactively engage with AI advancements through workshops, conferences, and online resources to stay informed and adept. This proactive stance will prepare the pharmacy sector in the MENA region to capitalize on AI’s benefits while navigating its challenges. Ultimately, our findings offer a critical foundation for refining pharmacy curricula, empowering graduates with the requisite AI skills, and fostering the responsible and strategic integration of AI in pharmacy practice.

## Supporting information

S1 Data The survey dataset for participants’ responses. (XLSX)
