# Correction: Knowledge, attitude and practice among pharmacy students and faculty members towards artificial intelligence in pharmacy practice: A multinational cross-sectional study

**DOI:** 10.1371/journal.pone.0336818

**Published:** 2025-11-13

**Authors:** Hisham E. Hasan, Deema Jaber, Samaa Al Tabbah, Nabih Lawand, Hana A. Habib, Noureldin M. Farahat

There is an error in affiliation 3 for author Nabih Lawand. The correct affiliation 3 is: Faculty of Medicine, Beirut Arab University, Beirut, Lebanon.
